# Bacterial Adaptation to Stress Induced by Glyoxal/Methylglyoxal and Advanced Glycation End Products

**DOI:** 10.3390/microorganisms13122778

**Published:** 2025-12-06

**Authors:** Dorota Kuczyńska-Wiśnik, Karolina Stojowska-Swędrzyńska, Ewa Laskowska

**Affiliations:** Department of General and Medical Biochemistry, Faculty of Biology, University of Gdansk, Wita Stwosza 59, 80-308 Gdansk, Poland; dorota.kuczynska-wisnik@ug.edu.pl (D.K.-W.); karolina.stojowska-swedrzynska@ug.edu.pl (K.S.-S.)

**Keywords:** glyoxal (GO), methylglyoxal (MGO), advanced glycation end products (AGEs), gut microbiota, glyoxalase pathway

## Abstract

Glyoxal (GO) and methylglyoxal (MGO) are highly toxic metabolic byproducts that induce carbonyl stress in bacteria and eukaryotes. Their accumulation in cells is linked to non-enzymatic glycosylation (glycation) of proteins, nucleic acids, and lipids, leading to the formation of advanced glycation end products (AGEs). In humans, AGEs are associated with several health problems, such as diabetes, Alzheimer’s disease, cancer, and aging. Recent studies indicate that, despite their short lifespan, bacteria are also affected by AGEs formation. In this review, we summarize the pathways and mechanisms that help bacteria cope with GO, MGO, and AGEs. We also discuss the impact of dietary AGEs on gut microbiota and the antibacterial activity of host-derived GO/MGO. Recent studies highlight three main areas for future research: the role of AGEs in dysbiosis, the regulation of protein activities by MGO/GO-dependent modifications, and the potential use of glyoxalase pathway inhibitors to combat pathogens. This last point is especially important due to the rising prevalence of multidrug-resistant strains and the failure of antibiotic therapies.

## 1. Introduction

**Figure 1:**
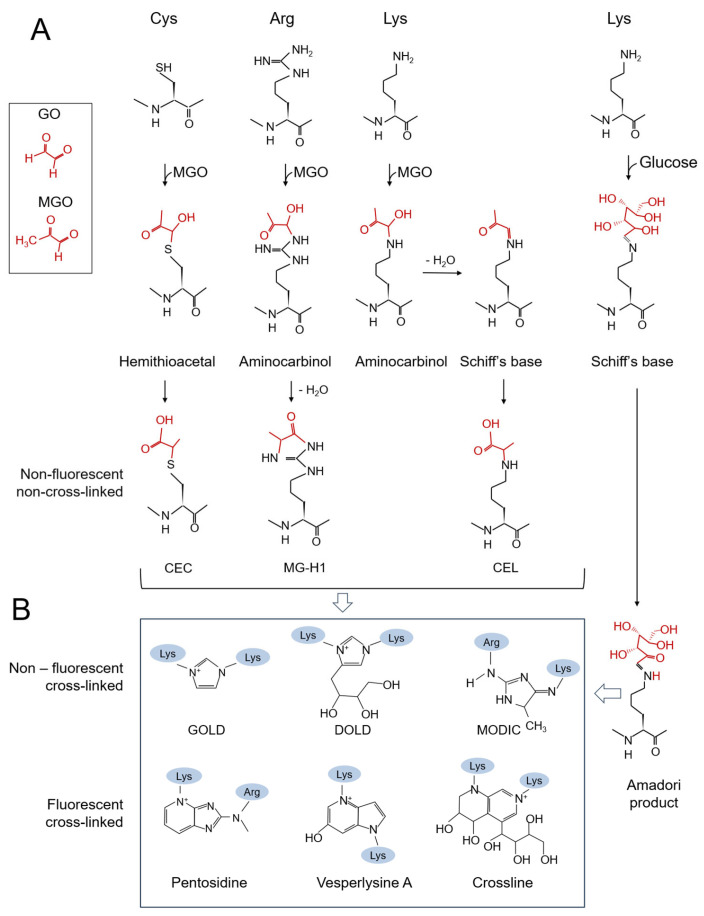
Glycation of proteins. (**A**) In the early stage of the Maillard reaction, the aldehyde form of monosaccharides or glycolytic by-products, such as MGO, reacts spontaneously with the thiol and amino groups of proteins. The resulting hemithioacetal, aminocarbinol, and Schiff’s bases are converted into more stable Amadori products and non-cross-linked AGEs. (**B**) Rearrangement of Amadori products (which leads to additional GO/MGO formation) and glycoxidation reactions create a highly diverse range of crosslinked AGEs, some of which are shown in the figure. CEL, carboxyethyl lysine; MG-H1, methylglyoxal-derived hydroimidazolone-1 (MGO forms with Arg two additional isomers, MG-H2 and MG-H3); CEC, carboxyethyl cysteine; GOLD glyoxal-derived lysine dimer; DOLD deoxyglucosone-derived lysine dimer; MODIC methylglyoxal-derived imidazolium cross-link.

Dialdehyde glyoxal (GO) and alpha-ketoaldehyde methylglyoxal (MGO) are toxic, highly reactive electrophile species (RES), produced as byproducts of the metabolism of carbohydrates, proteins, and lipids. The accumulation of GO and MGO, known as carbonyl stress, causes toxic effects through a series of non-enzymatic reactions that modify proteins, nucleic acids, and lipids, and ultimately leading to the formation of advanced glycation end products (AGEs) [1,2,3,4,5]. In the initial step of the so-called Maillard reaction, the carbonyl groups of reducing sugars or other aldehydes and ketones react non-enzymatically with free amino or thiol groups of proteins and nucleic acids [6]. The resulting adducts are then sequentially transformed into Schiff’s bases and Amadori products. The stable intermediates undergo cyclization and condensation, leading to the formation of AGEs (Figure 1). Depending on their chemical structure and ability to emit fluorescence, AGEs can be categorized into several groups: fluorescent and crosslinked, non-fluorescent and non-crosslinked, and non-fluorescent and crosslinked [2,6,7]. The formation of AGEs, particularly cross-linked products, can lead to proteotoxic effects, promoting or accelerating protein unfolding and single-strand breaks in DNA, resulting in increased mutation frequencies [4,8,9,10,11,12,13]. AGEs have been linked to various human degenerative diseases, including diabetes mellitus, Alzheimer’s disease, atherosclerosis, cancer and aging [3,9,14,15,16]. Since many AGEs are formed through the combination of oxidation and glycation reactions, glycation is interconnected with oxidative and nitrosative stress [5,17,18]. Because detoxifying GO/MGO requires NADPH and GSH (see Section 3), it may disrupt the redox balance and electron transport chain, leading to increased reactive oxygen (ROS) and nitrogen species (RNS) production. GSH also acts as a ROS/RNS scavenger; therefore, oxidative stress may temporarily hinder the detoxification of GO/MGO [19]. In addition, glycated residues in proteins are highly reducing and, in the presence of metals, generate superoxide radicals [20]. Studies on pathological conditions in humans indicate that AGEs increase ROS/RNS formation and impair the antioxidant defense system, whereas oxidative conditions trigger the formation of some AGEs [18].

Although AGEs occur across all three domains of life, they remain largely understudied in bacteria. Glycation products formed in *Escherichia coli* were reported for the first time by Mironova et al. [21]. AGEs were detected in recombinant human interferon gamma (rhIFN-g) produced in *E. coli* and in the total protein of *E. coli*. Glycation was initiated during normal *E. coli* growth and continued after protein isolation during long-term storage at −20 °C. Further experiments demonstrated that *E. coli* DNA also accumulates early- and late-glycation products under physiological conditions, predominantly during the stationary phase [22]. The aim of this review is to summarize the pathways and mechanisms that help bacteria cope with GO, MGO, and AGEs. The impact of dietary AGEs on the gut microbiota and the antibacterial activity of host-derived GO/MGO are also discussed.

## 2. Production and Toxic Effects of GO/MGO and AGEs in Bacteria

**Figure 2:**
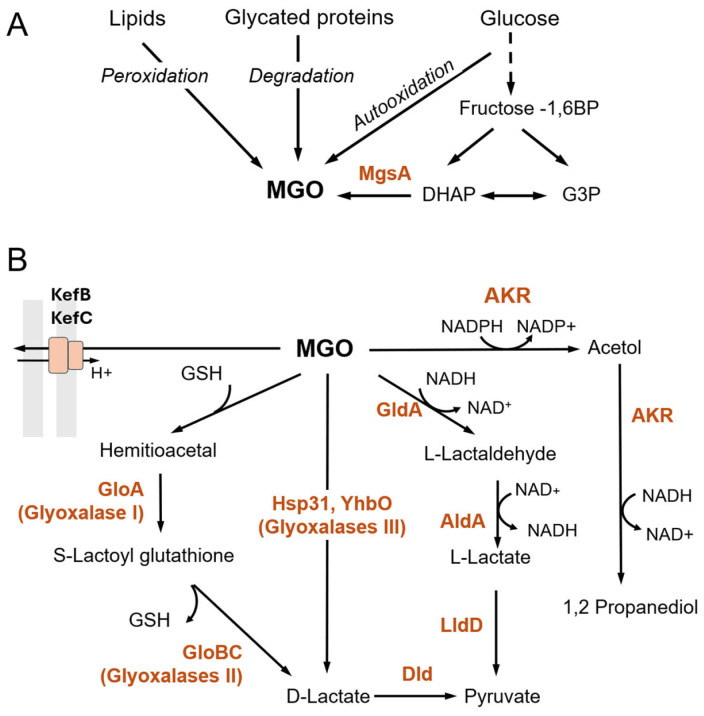
Formation and removal of MGO in bacteria. (**A**) The conversion of DHAP by the MGO synthetase MgsA is the primary source of MGO in bacteria. (**B**) MGO can be removed from the cell by the potassium efflux system, Kef, through the GSH-dependent pathway involving glyoxalases GloA, GloB, and GloC or by type III glyoxalases Hsp31 and YhbO, which lead to D-lactate formation that is then oxidized to pyruvate by Dld dehydrogenase. Other MGO detoxification pathways include GldA dehydrogenase and aldo-keto reductases (AKR). L-lactaldehyde is converted into pyruvate in two consecutive reactions catalyzed by lactaldehyde dehydrogenase AldA and lactate dehydrogenase LldDs. DHAP, dihydroxyacetone phosphate; G3P, glycerol-3-phosphate; GSH, glutathione.

The main source of MGO in bacteria and eukaryotes is the conversion of dihydroxyacetone phosphate by methylglyoxal synthetase (MgsA in *E. coli*) (Figure 2A) [15]. In bacteria, MGO production and its subsequent oxidation to pyruvate form a glycolytic bypass activated in response to phosphate depletion and triose phosphate accumulation. Thus, MGO synthesis may be beneficial as it enables the cell to sustain growth under imbalanced central metabolism [23,24]. GO and MGO can be formed directly from glucose via retroaldol condensation and autooxidation [25]. Other sources of GO/MGO include oxidation of lipids or DNA, glycine and threonine metabolism, and degradation of glycated proteins [1,15].

The Maillard reaction can occur in *E. coli* even under standard laboratory conditions, leading to reduced survival during the long-term stationary phase [26,27]. In aging *E. coli* cultures, carboxymethyl lysine (CML) and fluorescent AGEs are accumulated over time [27]. The addition of excess glucose or MGO to the medium results in a concentration-dependent increase in CML formation and a decrease in cell viability. Kramer et al. have shown that the composition of rich culture media used in laboratory experiments can cause significant changes in *E. coli* physiology—bacteria in very rich media experience increased oxidative stress due to the metabolism of excess nutrients [28]. This, in turn, results in higher glycation levels and mutation rates, ultimately leading to cell damage or death. Protein glycation can also occur during desiccation stress, a condition that bacteria often face in the natural environment [29]. Since water loss decreases fluidity, increases the concentration of macromolecules and destabilizes proteins [30,31,32,33], desiccation led to protein aggregation. Surprisingly, it was found that most cytoplasmic crosslinked AGEs were not trapped in protein aggregates but remained soluble [29]. It was concluded that protein aggregates served as a reservoir of partly active and native polypeptides protected against glycation, which can be rescued and refolded after rehydration and return to normal conditions. The highest level of CML was observed in the OmpC porin. Interestingly, CML was not found in another abundant *E. coli* porin OmpA. This result may be explained by the fact that lysine glycation depends on the secondary protein structure and the presence of specific residues or sequences (glycation hot spots) close to modified lysines [34,35]. The *E. coli* porins differ in structure: OmpC forms trimers of large 16-stranded β- barrels, whereas OmpA is a two-domain protein with a smaller 8-stranded β-barrel and a globular periplasm domain [36,37].

## 3. Detoxification of GO/MGO

The primary first line of defense against GO/MGO toxicity in Gram-negative bacteria is the activation of Kef systems (Figure 2B) [38,39]. The Kef monovalent cation, proton antiporters, which exchange intracellular K+ for external H+, are kept in a closed state by GSH and are triggered by GSH adducts formed in reaction with GO/MGO or other electrophiles. This process leads to the acidification of the cytoplasm, lowering the pH from 7.8 to 7.4. In *E. coli*, depending on the electrophile, one of the two main transporters, KefB or KefC, is activated in response to RES overload [40,41,42]. The exact mechanism explaining how acidification protects cells against GO/MGO is not fully understood; however, it is known that the reduction in cytoplasm pH prevents DNA damage by MGO, probably by activating DNA repair enzymes [43]. Cytoplasmic pH changes may be part of metabolic adaptation to stress, including resistance to antibiotics [44].

MGO can be removed from the cell by converting it into pyruvate via several pathways initiated by (1) MGO attaching to glutathione and hydrolyzing the resulting R-lactoyl glutathione to D-lactate, (2) directly converting MGO to D-lactate, and (3) reducing MGO to lactaldehyde followed by oxidation to L-lactate (Figure 2B) [45,46].

### 3.1. Glutathione-Dependent Pathway

GSH, the-L-glutamyl L-cysteinyl-glycine tri-peptide, is the most abundant low-molecular-weight thiol (0.1 mM–20 mM) present in all kingdoms of life (reviewed in [47]). It plays pleiotropic roles in metabolism, maintains cellular redox balance, and acts as a cofactor for various enzymes. GSH protects cells against oxidative stress by scavenging ROS and RNS and participates in the detoxification of harmful compounds, including heavy metals and MGO. In bacteria, GSH is synthesized in two consecutive steps catalyzed by glutamylcysteine synthetase and glutathione synthetase. The ratio of GSH to its oxidized form, a glutathione disulfide dimer (GSSG), determines the cell’s redox status. For example, in *E. coli* growing in standard LB medium, the GSH/GSSG ratio is 200 and decreases significantly under oxidative stress. GSSG removal from the cell involves GSSG reduction by glutathione reductase to GSH, ATP-dependent export, and GSSG degradation by peptidases [47,48].

In almost all organisms, MGO attaches to glutathione to form a hemithioacetal spontaneously [1]. The cyanobacterium *Synechocystis* sp. is an exception. One of the glutathione transferases, Sll0067 of the *Synechocystis* sp., is stimulated by S-D-lactoylglutathione and catalyzes the conjugation of MGO with GSH to initiate its elimination by glyoxalases [49]. It is worth noting that in photosynthetic organisms, MGO originates not only from the catabolism of sugars, amino acids, and lipids, like in heterotrophic organisms, but also from photosynthetic assimilation of CO_2_. In *E. coli*, the glutathione-dependent pathway involves type I glyoxalase GloA, which converts hemithioacetal into S-lactoylglutathione. S-lactoylglutathione is then transformed into D-lactate by the glyoxalase II isomers GloB and GloC [50,51,52,53]. A glutathione-dependent glyoxalase pathway is the main defense against MGO in *E. coli* and other Gram-negative bacteria. In Gram-positive Firmicutes, including *Bacillus subtilis*, an alternative thiol compound, bacillithiol (BSH) is used for MGO detoxification [54]. BSH is composed of L-cysteine linked to a glucosamine and malic acid. Similarly to the GSH-dependent pathway in *E. coli*, *B. subtilis* uses glyoxalase I (GlxA) and glyoxalase II (GlxB) to convert MGO into D-lactate. *B. subtilis* also has three homologs of glyoxalase III (YdeA, YraA, and YfkM) that convert MGO into D-lactate in a glutathione-independent way [54].

In *E. coli*, the *gloA* gene is transcribed together with the *nemRA* operon [52,55]. The *nemRA* operon encodes a repressor and the N-ethylmaleimide reductase, respectively, that are involved in cytosolic detoxification of electrophiles, including quinones and glyoxals. In the presence of electrophiles, a reversible disulfide bond is formed in NemR, resulting in lower DNA binding affinity and *nemA* derepression. The NemA is an FMN-containing enzyme that reduces various electrophiles, including quinones. The NemR repressor senses electrophiles by forming reversible disulfide bonds at Cys-21 and Cys-116, thereby altering its DNA-binding affinity. Thus, GloA, whose gene is located next to *nemRA* in the same operon, is part of the cytosolic system that maintains proper intracellular redox balance.

### 3.2. Glutathione-Independent Pathways

The glutathione-independent conversion of GO/MGO in *E. coli* is catalyzed by members of the DJ-1/ThiJ/PfpI superfamily, including Hsp31, YajL, YhbO, and ElbB [46,56]. Human DJ-1, an antioxidant and oxidative stress sensor, plays an important role in neuroprotective mechanisms. DJ-1 is associated with Parkinson’s disease as mutations in DJ-1 are responsible for approximately 1% of all early-onset Parkinson’s disease cases [57]. Bacterial DJ-1 homolog, Hsp31 (the product of the *hchA* gene), was previously reported to function as a heat-inducible holding chaperone that sequesters unfolded proteins and prevents their aggregation under prolonged, severe stress conditions [58]. The role of Hsp31 in acid stress resistance has also been reported [59]. YajJ, as a covalent chaperone, forms mixed disulfides with client substrates via its conserved Cys residues, protecting proteins against aggregation [60,61]. Further studies demonstrated that DJ-1 and its bacterial homologs may function as glyoxalases III that convert free GO and MDO to glycolate and lactate, respectively [46,56]. However, Richarme et al. proposed that DJ-1, Hsp31, YhbO and YajL are rather deglycases that repair proteins and nucleotides from endogenous glycation by GO and MGO. These results have been the subject of discussion [46,62,63,64,65,66]. It has been suggested that the removal of free MGO by DJ-1glyoxalase causes immediate decomposition of hemithioacetals due to the shift in equilibrium position, which may be mistaken for deglycase activity [64]. Choi et al. found that DJ-1 directly recognizes MGO but not MGO-intermediates [63].

Another detoxification pathway for MGO involves the GldA enzyme, which converts MGO into lactaldehyde [45]. Lactaldehyde is then oxidized by AldA dehydrogenase to produce lactate, which is further converted by LldD lactate dehydrogenase into pyruvate. The main role of GldA is to reduce dihydroxyacetone into glycerol, although it can also catalyze the reverse reaction—NAD-dependent oxidation of glycerol into dihydroxyacetone. Importantly, high levels of dihydroxyacetone are lethal to bacteria because it spontaneously converts to MGO [45,67]. Therefore, GldA prevents MGO accumulation not only directly but also by removing its precursor-dihydroxyacetone.

Additional key enzymes involved in GO/MGO detoxification are aldehyde and aldo-keto reductases (AKRs). Lee et al. observed that the GO sensitivity of a *gloA*-deficient *E. coli* strain was comparable to that of the wild-type strain, indicating that the glyoxalase system is not a main GO detoxification pathway [68]. Indeed, it was found that an NADPH-dependent aldehyde reductase, YqhD, facilitates the removal of GO by converting it to glycolaldehyde and then to ethadiol [68]. Four NADPH-dependent broad specificity AKRs have been identified in *E. coli*: YafB (DkgB), YqhE (DkgA), YeaE, and YghZ [69]. AKRs convert GO and MGO into glycolaldehyde and acetol, respectively [69,70]. Further studies demonstrated that although YgzH possesses MGO reductase activity, its main function is the detoxification of L-glyceraldehyde 3-phosphate by its reduction to L-glycerol 3-phosphate [71].

Bacterial species, including Gram-negative (*E. coli*, *P. aeruginosa*) and Gram-positive bacteria (*B. subtilis*, *Streptococcus* sp., *Listeria monocytogenes*, and *Mycobacterium tuberculosis*), produce homologs of glyoxalases and AKRs [54,72,73,74,75]. In vitro studies with purified enzymes or bacterial extracts indicate that they are involved in similar pathways (Figure 2). However, the exact functions of these homologs, their substrate specificities, and their roles in GO/MGO detoxification in vivo remain to be clarified. The problems may stem from discrepancies between in vivo and in vitro activities of some enzymes [72] and from the redundancy of AKR and DJ1 family proteins, which makes it difficult to obtain single knock-out mutants with visible phenotypes.

## 4. Detoxification of Amadori Products and AGEs in Bacteria

**Figure 3:**
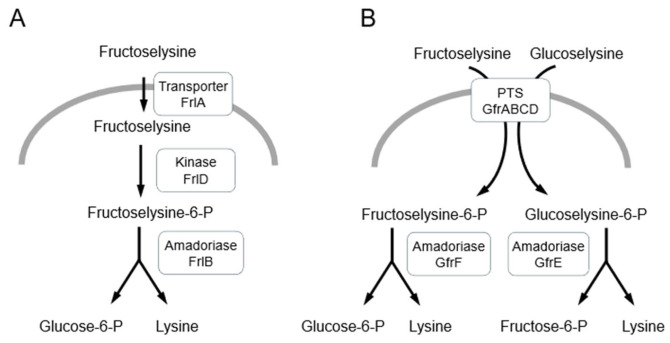
Examples of detoxification pathways for Amadori products in bacteria. Endogenous and exogenous Amadori products, fructoselysine and glucoselysine, are phosphorylated and then converted into glucose-6-phosphate (or fructose-6-phosphate) and lysine by specific amadoriases. (**A**) In *E. coli*, extracellular fructoselysine is transported by FrlA and then phosphorylated by FrlD kinase. (**B**) In *S. typhimurium* and *E. faecium*, Amadori products are phosphorylated during their transport by the phosphotransferase system (PTS) GfrABCD.

Decomposition of Amadori products by bacteria can be catalyzed by various enzyme groups. Deglycases comprise fructosamine kinases and amadoriases (FAD-dependent fructosyl amino acid oxidases). Fructosamine kinases phosphorylate the Amadori products before the cleavage [20,76,77]. In mammals, phosphorylation occurs at C-3, and the unstable product undergoes spontaneous degradation, whereas in bacteria, kinases phosphorylate at C-6; thus, the next step requires an additional deglycase enzyme—amadoriase—which catalyzes the oxidative degradation of Amadori products into the corresponding amino acid, glucosone, and hydrogen peroxide [20]. Apart from endogenous AGEs, bacteria can utilize external Maillard reaction products for ATP and metabolites synthesis (see Section 6). Two *E. coli* enzymes FrlD and FrlB have been shown to catabolize glycated lysines released upon digestion of food proteins in the human intestine [78]. Fructose-lysine is transported by FrlA into the cytoplasm and phosphorylated by the FrlD kinase in an ATP-dependent way. Next, FrlB catalyzes the hydrolysis of the resulting Nε-fructoselysine 6-phosphate to lysine and glucose 6-phosphate (Figure 3) [77]. Different bacteria may use modified kinase/deglycase systems to remove Amadori products. For example, in *S. typhimurium*, fructoselysine and glucoselysine are utilized through a mannose family phosphotransferase system (PTS) encoded by the *gfrABCD* operon. The fructose amines are phosphorylated during the transport and then cleaved by the GfrF or GrfE deglycases to produce lysine and glucose-6-phosphate or fructose-6-phosphate [79]. *Enterococcus faecium* produces GfrF, the FrlB homolog, and GfrE, the glucosamine-6-phosphate synthase homolog. GfrF converts reversibly fructoselysine 6-phosphate into glucose 6-phosphate and lysine, while GfrE catalyzes the formation of glucoselysine 6-phosphate from fructose-6-phosphate and lysine, and the reciprocal conversion of fructose 6-phosphate from glucoselysine 6-phosphate. The 6-phospho derivatives are presumably formed by the PTS encoded by *gfrA–gfrD* [76]. Recently, it has been demonstrated that phosphoglucose isomerase (Pgi) from *E. coli* possesses amadoriase activity [80]. The glycolytic enzyme Pgi catalyzes the isomerization of glucose-6-phosphate to fructose 6-phosphate in the second step of the Embden-Meyerhof-Parnas pathway. Boteva et al. found that Pgi degrades in vitro Amadori products, which are formed in chicken lysozyme and in native *E. coli* proteins. Since *E. coli* and human phosphoglucose isomerases exhibit 65% sequence identity and structural similarity, it has been proposed that this moonlighting enzyme may serve as a deglycase of glucose-6-phosphate-modified proteins in a broad range of organisms from bacteria to humans [80].

Intracellular proteolytic degradation of high-molecular-weight AGEs to low-molecular-weight products can facilitate the removal of AGEs from *E. coli* cells. Using selected knockout mutants and specific protease inhibitors, Cohen-Or et al. found that metalloproteases—but not ATP-dependent proteases, primarily responsible for protein quality control—are involved in the clearance of AGEs [81]. It was found that low-molecular-weight AGEs are secreted through efflux pumps in an energy-dependent manner [82]. Although the AGE-specific efflux pumps have not yet been identified, it is known that this process requires TolC. TolC is a versatile outer membrane protein that cooperates with numerous drug efflux pumps (AcrAB, AcrD, AcrEF, MdtABC, MdtEF, MacAB, EmrAB, EmrKY) involved in antimicrobial resistance or acid resistance (MdtABC, EmrAB) [83]. It was suggested that AGEs secreted by *E. coli* cells contribute to the inflammatory response in mammalian cell cultures, indicating that they may play a role in bacterial infections. It is plausible that host RAGE (Receptor for Advanced Glycation End-products) receptors may detect bacteria through secreted AGEs [82].

The instability of early glycation products, the complexity of reactions, and the diversity of structures pose significant challenges for detecting AGEs and understanding detoxification mechanisms in bacteria and other organisms. Studies on the glyoxalase versus deglycase activity of DJ-1 homologs show how difficult it is to assign a specific enzyme to a particular substrate. Since most AGE modifications in vivo remain undetected, it is not easy to identify all enzymes or mechanisms involved in removing Amadori products and AGEs.

## 5. Small Molecule Inhibitors of Glycation

A broad spectrum of small molecules with different mechanisms of action has been reported to inhibit glycation in humans [4,84]. Some of them, including trehalose, folic acid, carnosine and aminoguanidine, have been shown to protect bacteria against AGEs and improve their survival when added to the media [21,27,29]. Bacteria can produce folic acid and trehalose. Therefore, the protective effects of trehalose and folic acid may potentially be achieved through the upregulation of their synthesis. Trehalose, unlike reducing sugars, lacks a free aldehyde or ketone group, preventing it from initiating the Maillard reaction. It acts as a free radical scavenger and may stabilize proteins, inhibiting their unfolding and exposition of glycation hot spots [85]. Folic acid can modulate cellular glutathione levels, thereby affecting the protective glyoxylase system [27]. In addition, folic acid may react with reducing sugars [86], thereby limiting the rate of MGO formation via glycolysis. Recent studies demonstrated that folic acid binds human serum albumin (HSA) through hydrophobic interaction and hydrogen bond, preventing HSA glycation [87]. Carnosine, β-alanyl-L-histidine, is a naturally occurring dipeptide with several protective functions, including antioxidant, anti-inflammatory and anti-glycation activities. Carnosine is synthesized mainly in long-lived mammalian tissues, such as muscle and brain tissue. Multiple studies have shown that carnosine exerts positive effects on diabetes, cancer and neurodegenerative diseases (reviewed in [84,88]). The amino group and imidazole ring of carnosine may bind directly to MGO, GO and carbonyl groups in proteins treated with MGO [89]. Brownson and Higgins proposed that modification of carbonyl groups by carnosine may protect proteins from crosslinking and degradation. Aminoguanidine, in turn, inhibits the formation of AGEs by acting as a carbonyl and dicarbonyl trap. Aminoguanidine may react with carbonyl or α-hydroxycarbonyl sugars or intermediates to form a hydrazone, while its reaction with dicarbonyl compounds leads to the formation of a triazine [90]. Inhibitors of glycation can be particularly useful as protective agents during fermentation in nutrient-rich media when bacteria are exposed to carbonyl stress, or during long-term storage of protein samples, which can undergo progressive glycation even at freezing temperatures [21].

## 6. Gut Microbiota and Dietary AGEs

**Figure 4:**
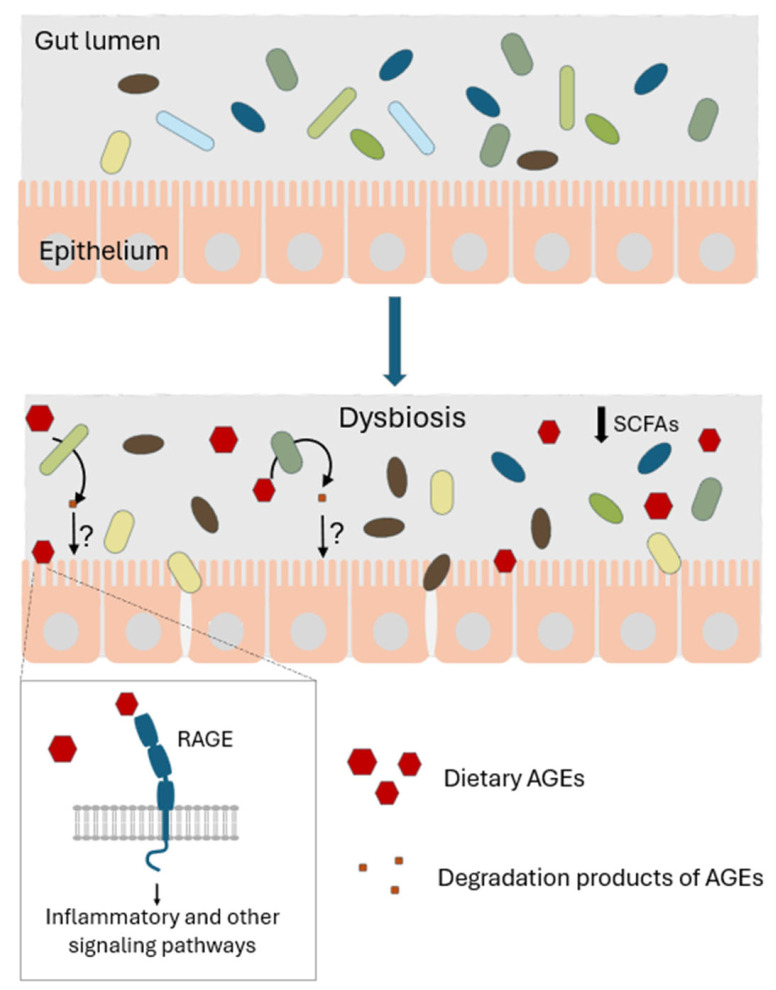
Influence of dietary AGEs on gut microbiota. Dietary AGEs are partly absorbed in the small intestine. Those AGEs that reach the large intestine affect microbiota composition, leading to dysbiosis. Growth of bacterial species that synthesize short-chain fatty acids (SCFAs), which are beneficial for gut health, is inhibited. Bacteria can degrade dietary AGEs; however, the effects of the resulting products on the microbiome, gut barrier, and inflammation pathways remain unknown. AGEs, through binding to RAGE, activate various intracellular signaling cascades, including proinflammatory and oxidative stress pathways.

AGEs accumulate in the body through endogenous synthesis and exogenous intake [91]. Endogenous AGEs, formed by posttranslational glycation of proteins, can be efficiently removed by autophagy and the ubiquitin-dependent 26S proteasome system or excreted. The balance between production and removal of endogenous AGEs is disrupted during various pathological states such as aging, hyperglycemia, and dyslipidemia. However, in healthy individuals, the main source of the total body burden of AGEs is food-derived (dietary) AGEs, mainly bound to proteins and formed during heating. Dietary AGEs play a significant role in gut microbiota dysbiosis (Figure 4), including decreasing microbiome diversity and increasing pathogens at the expense of beneficial species, leading to several human diseases such as hypertension, obesity, cancer, cardiovascular disease, and chronic kidney disease [92,93,94,95]. An increasing number of studies indicate that dietary AGEs can be degraded by gut microbiota, concomitantly modulating the microbiota composition [95,96,97,98,99,100]. Hellwig et al. found that several probiotic *E. coli* strains are able to degrade CML to biogenic amine- carboxymethylcadaverine and other defined metabolites [89]. Bui et al. (2019) demonstrated that intestinal bacteria, mainly *Oscillibacter* and *Cloacibacillus evryensis*, can degrade CML under anaerobic conditions. Carboxymethylated biogenic amines and carboxylic acids were identified as CML degradation products by *C. evryensis* [101]. Shi et al. analyzed the composition of gut microbiota during in vitro fecal fermentation with methylglyoxal-beta-lactoglobulin AGEs (MGO-β-LG AGEs) as the only nitrogen source [98]. At various time points of fermentation, a total of 187 strains able to metabolize AGEs were isolated. The composition of gut microbiota community changed over time. For example, at the initial stage of fermentation, a total of nine dominant bacterial genera were identified. However, after 8 h of fermentation, dominant bacterial genera comprised only *Klebsiella* and *Escherichia-Shigella*. A total of 18 and 129 different types of AGE-modified peptides were identified by high-resolution mass spectrometry before and after fermentation, respectively. This result clearly indicated that bacteria catabolize MGO-β-LG AGEs [98]. Other studies have shown that chronic exposure of mice to dietary AGEs led to CML deposition, upregulation of RAGE in the ileum and systemic inflammation. These effects at least partly resulted from a reduction in *Anaerostipes*, a species that produces butyrate, a short-chain fatty acid that helps maintain the gut barrier and exerts anti-inflammatory effects [100]. Further research is necessary to clarify the fate of AGE-derived metabolites. It remains unknown whether they are released into the bloodstream, influence the gut barrier, or play a role in the pathophysiological outcomes linked to dietary AGEs.

It should be noted that several reports have suggested that dietary AGEs have beneficial effects on gut microbiota (reviewed in [95]). For example, the milk proteins β-lactoglobulin and casein, conjugated with galactose and lactose via the Maillard reaction, were fermented by *Lactobacillus* and *Bifidobacterium*, thereby boosting their growth [102], while the administration of glycated fish proteins enhanced microbiota diversity and increased the abundance of bacterial species that produce butyrate in rats [103].

## 7. Host-Derived MGO/GO as an Antibacterial Agents

### 7.1. MGO/GO Production in Mammalian Cells

In mammalian cells, most MGO (~90%) is produced endogenously during glycolysis, primarily from glyceraldehyde-3-phosphate and dihydroxyacetone phosphate [104]. MGO is also produced during the breakdown of threonine through aminoacetone oxidation by semicarbazide amine oxidase (SSAO), and during ketone body breakdown via acetone oxidation catalyzed by cytochrome P450 2E1. These pathways generate relatively low MGO levels, except when ketone body levels rise, such as in diabetic ketoacidosis, fasting, or a low-calorie diet [104]. Degradation of glycated proteins accounts for about 7% of total MGO levels. Typical concentrations of GO and MGO are 50–150 nM in human plasma and 1–4 μM in mammalian cells [104]. The intracellular concentration of MGO could be elevated by increasing the activity of glycolysis due to excessive glucose or fructose diet. This, in turn, leads to increased expression of RAGE, which mediates the transport of AGEs into the cell and activates proinflammatory factors, generating ROS [92]. Under normal conditions, 98% of endogenous MGO is detoxified by the glyoxylase system [94]. Despite MGO’s high toxicity, the human body may utilize its production as an innate immune antimicrobial effector during pathogen infection [105].

### 7.2. Glyoxalase Pathway as a Way to Evade Host Response

**Figure 5:**
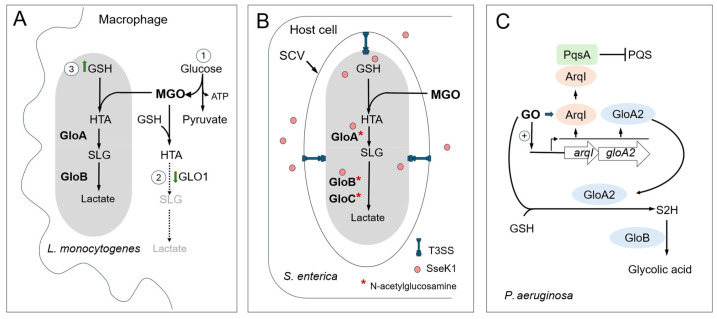
Host-derived MGO/GO as antibacterial molecules and bacterial counter-adaptations. (**A**) During bacterial infection, the level of MGO in macrophages increases due to a metabolic shift toward anaerobic glycolysis, which promotes MGO production. Downregulation of the host detoxification system, including glyoxalase 1 (GLO1), contributes additionally to the enhanced MGO synthesis. Upon entry into macrophages, *L. monocytogenes* upregulates GSH production to efficiently eliminate host MGO through the formation of HTA, which is then converted to lactate by glyoxalases I (GloA) and II (GloB). (**B**) In Salmonella enterica, the glyoxylase pathway and the T3SS system are connected by the SseK1 glycosyltransferase. SseK1 is one of the three SseK glycosyltransferases that act as effectors of T3SS, modifying various host protein substrates with N-acetylglucosamine to decrease host inflammatory responses. SseK1 also modifies bacterial glyoxalases GloA, GloB, GloC, and the deglycase YajL, thereby increasing their activities and host-derived MGO detoxification. (**C**) Hypothetical model of a GO-specific signaling pathway in *P. aeruginosa*. GO triggers a two-gene operon that includes arqI and gloA2. ArqI inhibits the production of PQS quorum-sensing signals by directly interacting with PqsA, the first enzyme in the PQS biosynthesis pathway. GO is converted into glycolic acid through a GSH-dependent pathway involving GloA2 and GloB glyoxalases. GO, glyoxal; GSH, glutathione; HTA, hemithioacetal; MGO, methylglyoxal; SCV, Salmonella-containing vacuole; SLG, S-lactoyl-glutathione; S2H, S-2-hydroxyethylglutathione; PQS, Pseudomonas Quinolone Signal.

Pathogens capable of effectively eliminating external MGO are more likely to evade the host’s antibacterial defenses [73,74,75,106,107]. Since MGO reacts with guanine bases in DNA, increasing mutation rates in pathogens, its detoxification is essential to maintain genomic stability during infection. An example of a bacterium that employs glyoxylase pathways to evade the host defense system is a facultative intracellular pathogen, *Listeria monocytogenes* (Figure 5A) [83]. Upon entry into host cells, *L. monocytogenes* upregulates glutathione synthase GshF and GSH production, which allosterically activate the virulence regulator PrfA. Notably, the expression of *gshF* and virulence genes is activated by host-derived MGO [73]. Further studies revealed that during bacterial infections, activated macrophages produce ATP mainly through increased glycolysis rather than oxidative phosphorylation, even in the presence of oxygen. This metabolic shift in macrophages promotes MGO production, accompanied by downregulation of the host detoxification system, including glyoxalase 1 (GLO1) [73,105,108]. The *L. monocytogenes* lacking *gloA* exhibits attenuated virulence and increased mutation rate during intravascular infection in mice [73]. On the other hand, decreased MGO synthesis in mice results in enhanced survival of the pathogen [108]. The glyoxalase pathway also plays a crucial role during Group B *Streptococcus* (GBS) bloodstream infection. It was demonstrated that GBS infection increases intracellular MGO in neutrophiles and GloA contributes to GBS survival against neutrophiles in vivo and in vitro [74].

In *Salmonella enterica*, the glyoxylase pathway and the type III secretion system (T3SS) are interconnected by the SseK1 glycosylotransferase (Figure 5B). SseK1 is one of the three SseK glycosyltransferases that are effectors of T3SS2, modifying host protein substrates with N-acetylglucosamine on specific arginine residues. El Qaidi et al. reported that in *S. enterica*, the enzymatic activities of glyoxylases GloA, GloB, GloC, and deglycase YajL are enhanced after glycosylation of specific arginine residues by SseK1 [109]. The glycosylation increased resistance to MGO and improved the repair of MGO-damaged proteins. Therefore, SseK1 affects the host immune response not only by inhibiting NF-κB signaling [110] but also by enhancing the detoxification of MGO [109]. Recently, Corcoran et al. described the GO-mediated signaling pathway that enables *Pseudomonas aeruginosa* to survive in blood-rich organs [75]. It was found that GO induces a two-gene operon in *P. aeruginosa*, consisting of *gloA2*, which encodes a glyoxalase, and *arqI* (Aldehyde Responsive Quorum-Sensing Inhibitor), which encodes an ABM domain protein (Figure 5C). In the presence of GO, ArqI localizes to the flagellar pole and interacts with PqsA, the initial enzyme involved in the synthesis of the major quorum-sensing Pseudomonas quinolone signal (PQS) molecule. This may consequently lead to the blockage of PqsA-controlled processes such as iron uptake, biofilm formation, and immune modulation. Surprisingly, the conserved Arg49 residue in ArqI undergoes unusual post-translational modification by GO [75]. The mutant lacking the *arqI-gloA2* operon lost viability in blood-rich organs, confirming that GO-mediated signaling contributes to evasion of the host immune response.

### 7.3. Glyoxalase Pathway and Interspecies Competition in Oral Streptococci

Recent studies by Zeng et al. revealed that the glyoxalase pathway in oral streptococci contributes to interspecies competition between a caries-associated pathogen *Streptococcus mutans* and commensal *Streptococcus sanguinis* [106]. *S. mutans* could outcompete commensal streptococci in the presence of MGO due to differences in resistance levels and gene regulation. *S. mutants* displayed a greater MGO tolerance than *S. sanguinis*, but when glyoxalase I (Lgul) was missing in both strains, MGO tolerance was comparable. In addition, MGO induced the expression of *lguL* in *S. mutants*, but not in *S. sanguinis*. It was also shown that streptococci possess an additional glyoxalase gene, *gloA2*, which is necessary for the degradation of RES derived from fructose-1-phosphate [106]. It is worth noting that, when metabolized as fructose-1-phosphate, fructose can bypass a regulatory point in glycolysis—the phosphofructokinase that uses fructose-6-phosphate as a substrate—leading to faster generation of RES. The concentration of MGO in saliva increases significantly from 0.19–0.26 μM to 1.32–1.42 μM in individuals with type II diabetes [111]. Under these conditions, MGO detoxification becomes crucial for streptococci, which also favors caries-associated pathogens.

These multiple examples strongly suggest that inhibiting the glyoxalase pathway could be an effective strategy to boost the host’s antibacterial response and effectively fight pathogens.

## 8. Concluding Remarks and Future Perspectives

The mechanisms protecting cells against glycation are highly conserved among species; therefore, an increasing number of studies on bacteria may provide details that help to understand different aspects of AGE-related stress in other organisms. MGO/GO and AGEs are generally associated with toxic effects, cellular deterioration, and loss of viability. However, MGO/GO production in bacteria is inevitable, especially in glucose-rich media. Moreover, MGO synthesis provides a bypass pathway for glycolysis when triose phosphate accumulates in excess [23,24]. Takeuchi proposed that the generation of AGEs, which do not exert direct cytotoxic effects, such as CML, pentosidine, and pyrraline, is a defense mechanism that traps and detoxifies the end products of glycation/carbonyl stress, in contrast to toxic AGEs, which promote the onset of lifestyle-related diseases [112]. Although this concept cannot be directly implemented in bacteria, it can be speculated that some proteins (particularly those that are unfolded and expose glycation hot spots) can be marked through the Maillard reaction, trapped as separate crosslinked deposits and/or degraded and exported outside the cell. In this way, the cell can eliminate both MGO/GO and proteins damaged by RES or other stresses. Despite significant progress in understanding the effects of glycation in bacteria, there are still research gaps that need further investigation: identification and quantification of bacterial AGEs formed in vivo, the structure of crosslinked products, and the regulation of detoxification pathways.

Although it is generally accepted that protein glycation causes loss of function, recent studies have revealed that GO/MGO-dependent posttranslational modifications may regulate the activities of some proteins. As mentioned in the previous section, Arg1 modification by GO triggers a GO-specific response in *P. aeruginosa* [75]. Another example is the MGO-dependent activation of Tle, the type VI secretion system (T6SS) lipase effector from *Enterobacter cloacae* [113]. The toxic effector Tle, together with its cognate immune protein Tli, is involved in interbacterial competition. In Tle, the crosslink MODIC (methylglyoxal-derived imidazolium crosslink), formed between an arginine and a lysine residue, links the most distant N-terminal and C-terminal helices. This post-translational modification, which probably stabilizes the protein structure, is required for Tle phospholipase activity [113]. Further in silico studies indicate that a similar modification might occur in pore-toxin colicin Ia of *E. coli* [114]. The authors suggest that more proteins may undergo MGO/GO-dependent crosslinking; however, because MODIC/GODIC are unstable, their detection is highly challenging.

The interplay between dietary AGEs and gut microbiota requires further research. An increasing number of studies report that intestinal bacteria can consume exogenous free or protein-bound AGEs. However, it remains unclear whether AGE-derived metabolites are released into circulation, affect the gut barrier, or have any impact on pathophysiological outcomes related to dietary AGEs. In general, excessive dietary AGEs intake is linked to changes in gut structure, resulting in increased gut barrier permeability dysfunction, altered enteric neuron expression, inflammation and reduced gastrointestinal motility [100,115,116]. Gut motility can be decreased due to high blood glucose level (which also leads to enhanced glycation) and some medications such as GP-1 agonists used to treat type 2 diabetes and obesity [99,117]. This, in turn, delays the removal of AGEs from the gut and promotes further glycation reactions from precursors, forming a vicious cycle that may impact the microbiome composition. Although evidence showing that dietary AGEs cause dysbiosis is prevalent in the recent literature, there are several reports describing the beneficial effects of AGEs on the gut microbiota [95].

An important and intriguing area of research is the study of bacterial glyoxalase inhibitors as antibacterial agents. Several reports have shown that disrupting bacterial glyoxalase systems increases the sensitivity of pathogens to host MGO [73,74,75,106,107,108]. This approach could serve as a complementary strategy to antibiotics, especially against multidrug-resistant pathogens. *E. coli* GloA and human GLO1 share only 34% identical sequences. There are also differences in the active sites of GloA and GLO1 [51]. Therefore, designing GloA-specific inhibitors that do not affect the human homolog is feasible. The risk of developing resistance to GloA inhibitors seems to be lower than that associated with traditional antibiotics, in which horizontal gene transfer primarily contributes to the dissemination of antimicrobial resistance genes. Nevertheless, there are at least two possibilities: spontaneous *gloA* mutations that decrease the glyoxalase’s affinity for the inhibitor, or upregulation of alternative pathways that eliminate MGO across the entire bacterial population. To limit the adverse impact of the inhibitor(s) on the entire microbiome, designing a molecule that selectively binds only the glyoxalase of the targeted pathogen will be necessary.

## Data Availability

No new data were created or analyzed in this study. Data sharing is not applicable to this article.

## Abbreviations

AGEs	Advanced Glycation End products
AKR	Aldo-keto reductase
CEC	Carboxyethyl cysteine
CEL	Carboxyethyl lysine
CML	Carboxymethyl lysine
DHAP	Dihydroxyacetone phosphate
DOLD	Deoxyglucosone-derived lysine dimer
G3P	Glycerol-3-phosphate
GO	Glyoxal
GOLD	Glyoxal-derived lysine dimer
GSH	Glutathione
HSA	Human serum albumin
MG-H1	Methylglyoxal-derived hydroimidazolone-1
MGO	Methylglyoxal
MODIC	Methylglyoxal-derived imidazolium cross-link
PTS	Phosphotransferase system
RAGE	Receptor for Advanced Glycation End-products
RES	Reactive electrophile species
ROS	Reactive oxygen species
SCFAs	Short-chain fatty acids
